# Chemical Characterization of Coffee Husks, a By-Product of *Coffea arabica* Production

**DOI:** 10.3390/foods10123125

**Published:** 2021-12-16

**Authors:** Lais B. Cangussu, Jean Carlos Melo, Adriana S. Franca, Leandro S. Oliveira

**Affiliations:** 1 Graduate Program in Food Science, Universidade Federal de Minas Gerais, Av. Antônio Carlos, 6627, Belo Horizonte 31270-901, MG, Brazil; lai.sbc1@hotmail.com (L.B.C.); jeancarlosmelo1@hotmail.com (J.C.M.); leandro@demec.ufmg.br (L.S.O.); 2 Department of Mechanical Engineering, Universidade Federal de Minas Gerais, Av. Antônio Carlos, 6627, Belo Horizonte 31270-901, MG, Brazil

**Keywords:** agri-food by-products, dietary fiber, bioaccessibility, non-extractable phenolics, polysaccharides

## Abstract

Coffee husks are a major by-product of coffee production and are currently being underutilized. The aim of this work was to chemically characterize coffee husks to allow for an adequate evaluation of their potential for valorization. Blanched and non-blanched coffee husks were characterized for extractable and non-extractable phenolics, caffeine, trigonelline content, and for their polysaccharide and proximal composition. The total, soluble and insoluble fiber contents were determined, together with the husks’ technological properties. Antioxidant activity and bioaccessibility of phenolic compounds of coffee husks were evaluated. Two types of husk were studied: one comprised mostly of outer skin and pulp (CH1); and other comprised mostly of parchment (CH2). Blanching had positive effects on non-extractable phenolics, chlorogenic acid and on the bioaccessibility of phenolics, promoting small reductions in extractable phenolics, protocathecuic acid, caffeine and trigonelline contents. Blanched CH1 presented more appropriate properties than CH2 for potential applications in food. It also presented better antioxidant, hydration, and oil holding properties than those of other agri-food by-products. Tentatively identified polysaccharides included galactomannans, arabinogalactans type II, pectin and cellulose.

## 1. Introduction

Coffee is one of the main commodities in the world in terms of revenue, second only to petroleum, being consumed by approximately a third of the world population [1,2]. Brazil, Vietnam, Colombia and Indonesia are the largest producers in the world [2]. Coffee processing generates large amounts of by-products. Dry processing is the most common technique applied to coffee fruits [2], with main by-product being comprised of dried pulp and parchment and it is herein denominated coffee husks (CH). Dry processing generates approximately 1 kg of CH per kg of coffee beans produced [3]. The adequate allocation of these by-products contributes to a sustainable production, since these can cause serious environmental problems due to their content of highly bioactive compounds such as caffeine and trigonelline [4].

Coffee production by-products have been studied since the beginning of the twentieth century, mostly for applications as ingredients in animal feed and composting [3]. However, throughout the majority of the 20th century studies on such by-products were rather scarce and only recent have studies on these by-products as precursor materials to produce value-added products regained momentum [5,6,7,8,9]. A few products containing coffee by-products are already being commercialized or are awaiting approval for commercialization as novel foods around the world as reviewed by Klingel et al. [10]. To define suitable processes for valorization of coffee husks, it is necessary to properly physicochemically characterize this by-product. Proximate compositions of coffee husks have been scarcely published in the literature [3,6,11] with a rather large variability of the individual values for lipids (0.5–3%), protein (7–17%), ash (3–7%) and carbohydrates (16–85%) contents. A large variability is also observed for the published values for cellulose (14.7–46.1%), hemicellulose (10.2–29.7%) and lignin (10.1–34.2%) contents [5,12,13,14]. Aside from inherent agronomical and processing variabilities, the major reason for the wide range of values for each class of compound is the lack of a proper definition of what is termed ‘coffee husk’. The coffee fruit comprises several layers: outer skin (peel); pulp; mucilage; parchment; silverskin; and beans. When coffee is dry processed, the major wastes generated are the husks, which comprise dry pulp and parchment (including outer skin and mucilage). In wet processing, there are two separate wastes, generated in distinct steps of the processing: The wet pulp (including the outer skin); and the dry parchment (including a portion of the mucilage).

Extractable phenolics and other bioactive compounds were the classes of compounds more minutely studied in coffee husks, pulp and parchment, with identification and quantification of individual compounds, such as chlorogenic, ferulic and protocatechuic acids, rutin, catechin, epicatechin, anthocyanins and caffeine [15,16,17,18,19].

Polyphenols have received considerable interest due to their potent antioxidant activities [20] and are classified according to their structure as extractable phenols (EP) and non-extractable phenols (NEP), with the latter labeled macroantioxidants. Vegetable matrices, such as agri-food by-products, usually have higher contents of NEP than of EP [21]. However, NEP are hardly removed from the vegetable matrix when exposed to aqueous or organic solvents because they are entrapped in the matrix, strongly attached to polysaccharides and proteins [22,23]. The exploitation of the NEP fraction of agricultural by-products could allow surplus revenue for agricultural producers as the by-product is valorized. No studies on the non-extractable phenolics of coffee husks were found in the literature and the bioaccessibility of these by-products’ bioactive compounds has not been evaluated to allow for a definition of a suitable application in the food industry. Furthermore, the polysaccharide fraction of coffee husks was not properly characterized, with published data restricted to sucrose and total reducing sugars contents [3,5,6].

To evaluate the potential of minimally processed coffee husks, in the form of flour, as a functional food ingredient, there is a need to determine the bioaccessibility of its bioactive compounds. Hence, this study aimed at contributing to the evaluation of coffee husk flours as a potential source of dietary fibers and bioactive compounds by tentatively characterizing their polysaccharide fractions, by determining the contents of bioactive compounds, such as caffeine, trigonelline and phenolic compounds, and by investigating the bioaccessibility of the associated bioactive phenolic compounds.

## 2. Materials and Methods

### 2.1. Reagents

Standards for chromatography analyzes (ferulic acid, chlorogenic acid, caffeine, monosaccharides, D-allose) were obtained from Sigma-Aldrich (St. Louis, MO, USA), except protocatechuic acid, trigonelline (USP, São Paulo, Brazil) and D-(+)-xylose (Supelco, Bellefonte, PA, USA).

### 2.2. Flours Preparation

Two samples of husks, CH1 and CH2, obtained by dry processing of Bourbon Arabicas were used in this study: CH1, comprised of 80% peel and pulp and 20% parchment, was provided by the Coffee Industry Syndicate of the State of Minas Gerais (Belo Horizonte, Brazil); and CH2, comprised of 28% peel and pulp and 72% parchment, were provided by the Santa Inês Farm (Carmo de Minas, MG, Brazil). Husks were stored in plastic containers at 20 °C. Half the samples were blanched at 90 ± 2 °C for 1 min. The blanched (CH1b and CH2b) and unblanched (CH1 and CH2) samples were dried in a convective oven for 6 h at 60 ± 2 °C and, subsequently, ground, graded in a 35-mesh sieve (Tyler), and stored at −18 °C.

### 2.3. Extractable and Non-Extractable Phenolics

Half a gram of husk powders was placed in 50 mL Falcon tubes and extraction of phenolics was performed by adding 20 mL of methanol (50% *v/v*) and 20 mL of acetone (70% *v/v*). After each extraction, the samples were centrifuged at 3500× *g* rpm for 15 min. The supernatants, comprising total extractable phenolics (TEP), were combined and the volume made up to 50 mL with distilled water [21]. The residues were dried at 35 °C for 17 h and used in the determination of non-extractable phenolics (NEP).

Extractable phenolics were evaluated by the Folin–Ciocalteu method. 1 mL of extracts was combined with 1 mL of Folin-Ciocalteu reagent solution at a 1:3 ratio, 2 mL of sodium carbonate (20% *m/v*), and 2 mL of distilled water. Samples were stirred and let to rest for 90 min in the dark. Absorbance values were measured at 700 nm in a UV-VIS spectrophotometer (Micronal, São Paulo, Brazil) [21]. A calibration curve was built employing gallic acid and results expressed as gallic acid equivalents (mg GAE/g dry matter). Folin–Ciocalteu reagent reacts with reducing compounds, hence, quantification of phenolics by High Performance Liquid Chromatography (HPLC) was also carried out in our study. However, since this reagent/method has been employed for so long, not only for coffee and its by-products, but also for a variety of other agri-food by-products, it was used in this study to allow for comparison of the herein obtained results with those of other by-products from other studies. Also, aside from the not easily accessible reducing ends of long chain polysaccharides, there was no expectation of reducing compounds other than polyphenols (e.g., ascorbic acid, tocopherol and others) being present in significant amounts in coffee by-products.

Dry residues from the TEP extraction were mixed with 10 mL of n-butanol-HCl (95:5 *v/v*) containing 0.7 g/L of FeCl_3_ and kept at 100 °C for 50 min. The mixtures were centrifuged, and supernatants collected. Samples were washed twice with 5 mL of n-butanol-HCl (95:5 *v/v*) containing 0.7 g/L of FeCl_3_, centrifuged again, and supernatants collected. Volume was completed to 25 mL with butanol-HCl solution. Then, absorbance values were measured at 450 and 550 nm in a UV-Vis spectrophotometer. The sum of absorbance values was plotted against NEP concentration. The spectrophotometer was reset to zero with a white solution of n-butanol-HCl (95:5 *v/v*) containing FeCl_3_. Proanthocyanidin obtained from carob pods (*Ceratonia siliqua* L.) was used as standard [22]. Carob pods contain large amounts of proanthocyanidins and thus were utilized in our study as a source of this polymeric material, which was isolated using a variety of solvents (acetone, petroleum ether, methanol) assisted by ultrasonication, purified by chromatography (successive elution with methanol-water and acetone-water). The purity of the isolated proanthocyanidins was determined to be 76.5%.

In vitro digestion of the flour was evaluated in oral, gastric, and small intestinal phases, according to Dutra et al. [24]. 500 mg of husks flour were mixed with 12.5 mL saline solution (0.05 g/mL of Na_2_HPO_4_, 0.004 g/mL of KH_2_PO_4_, 0.16 g/mL of NaCl and 0.17 g of α-amylase). The samples were shaken at 95 rpm in an orbital incubator for 10 min at 37 °C. Subsequently, the mixtures were acidified (pH 2.5) with HCl 3 M, and 5 mL of pepsin preparation was added (13 mg of porcine pepsin in 5 mL of HCl 0.1 M). For gastric digestion simulation, samples were incubated for 1 h at 37 °C under agitation at 95 rpm and subsequently cooled in an ice bath. For the simulated small intestinal digestion, pH was adjusted to 7.5 with NaHCO_3_ 1 M, followed by addition of 5 mL of NaHCO_3_ 1 M containing 87 mg of pancreatin and 7 mg of bile salts. The mixture was incubated at 37 °C for 2 h under agitation, centrifuged at 3500× *g* rpm for 10 min, the supernatant was collected, and the volume was made up to 50 mL with distilled water. The extracts were used to quantify total extractable phenolics as previously described. The bioaccessibility percentage (% Bio) was calculated by:

(1) $$\%\;Bio=\frac{b\times 100}{a}$$

where *b* is the quantity of phenolics released after in vitro digestion and *a* is the quantity of phenolics prior to in vitro digestion.

### 2.4. Identification and Quantification of Phenolic Compounds by High-Performance Liquid Chromatography (HPLC)

Phenolic compounds extracts were obtained according to Dutra et al. [24]. Before extraction, chloroform was used to degrease the samples. 0.15 g of flour were extracted using different methanol concentrations (10 mL at 50%, 10 mL at 75%, and 5 mL at 100%) in a Dubnoff bath at 65 °C for 1 h. After each extraction, samples were centrifuged at 3500× *g* rpm for 15 min and supernatants combined, with volume completed to 25 mL with methanol 100%. Filtered samples were injected in a Prominence HPLC system (Shimadzu, Kyoto, Japan), using a Photodiode Array (PDA) detector (203–325 nm) and a Shimadzu C18 column (4.6 μm × 150 mm) (Shimadzu, Kyoto, Japan) at 50 °C. The mobile phase consisted of water and acetonitrile (92.6:7) containing 0.4% phosphoric acid (phase A) and acetonitrile containing 0.4% phosphoric acid (phase B) at a flowrate of 1.2 mL/min. Gradient elution was performed as follows: 0–8 min, 1–3% linear B; 8–12 min, 3–8% linear B; 12–15 min, 8–10% linear B; 15–20 min, 10–15% linear B; 20–25 min, 15–40% linear B; 25–30 min, 40–80% linear B; 30–35 min, 80–95% linear B; 35–35.1 min, 95–1% linear B and 35.1–42 min. –1% B isocratic. Phenolic compounds were identified by retention times and UV spectra of respective standards. Quantification was based on the standard curves.

### 2.5. Quantification of Caffeine and Trigonelline

Extraction of trigonelline and caffeine was conducted according to Perrone et al. [25]. We mixed 10 mL of boiling water with 0.1 g of sample. Samples were conditioned in a Dubnoff bath at 100 °C for 10 min under agitation. The mixtures were centrifuged for 10 min and supernatants collected. The extraction was repeated, and volume completed to 50 mL. Extracts were diluted (0.0004 g/mL), filtered (0.20 μm filters) and analyzed by HPLC using a PDA detector (264 nm for trigonelline; 272 nm for caffeine) and a Shimadzu C18 5 μm (4.6 μm × 150 mm) column at 18 °C. Mobile phases were water:methanol (95:5) for trigonelline and methanol:water (40:60) for caffeine, at a flowrate of 1 mL/min. Identification of caffeine and trigonelline was based on retention time and UV spectrum of individual standards, and quantification was based on calibration curves of respective standards.

### 2.6. Monosacharides Profile

Neutral monosaccharides composition was determined by gas chromatography, according to Leão et al. [26] 5 mg of samples were hydrolized with 0.5 mL of trifluoroacetic acid (2 mol/L). Subsequently, the resulting monosaccharides were reduced using 1 mL of sodium borohydride 0.5 mol/L in dimethylsulfoxide and derivatized to alditol acetates by adding 2 mL of acetic anhydride and 200 µL of 1-methylimidazole. Alditol acetates were extracted using 1 mL of dichloromethane and analyzed in a gas chromatograph (Varian 3900, Palo Alto, CA, USA) with Flame Ionization Detector (FID) and BPX-70 capillary column (30 m × 0.32 mm × 0.25 μm). Temperatures of the detector and injector were 280 and 230 °C, respectively. Carrier gas was nitrogen with flowrate of 1.5 mL/min for 38 min (30 s at 38 °C, temperature increased to 170 °C at a rate of 50 °C/min, then increased to 230 °C at a rate of 2 °C/min, and finally maintained for 5 min). Identification of the monosaccharides was undertaken using the respective standards. The relative molar ratio of the monosaccharides was calculated with respect to allose, employed as internal standard.

### 2.7. Chemical, Physical and Technological Properties

Proximate composition of the flour was evaluated by AOAC methods [27]. Moisture contents were determined after sample drying at 105 °C, until constant weight. Fat contents were determined by Soxhlet method after extraction with petroleum ether (AOAC method 4.5.05). Ash contents were determined by burning at 550 °C until obtaining white ashes (AOAC method 942.05). Crude protein was determined by the Kjeldahl method (AOAC method 960.52) using the 6.25 factor with subtraction of caffeine and trigonelline contents. Total dietary fiber (TDF) was determined by enzymatic–gravimetric method (AOAC methods 985.29 and 960.52). Samples were mixed with α-amylase at 100 °C for 15 min and digested with pepsin and pancreatin at 40 °C, for 60 min each. Insoluble dietary fiber (IDF) was filtered and washed with hot distilled water. Ethanol 95% at 60 °C was added to the filtrate and allowed to rest for 60 min to precipitate the soluble dietary fiber (SDF). IDF and SDF residues were dried at 105 °C for 16 h. TDF corresponded to the sum of IDF and SDF. Pectin content was determined by extraction with HCl followed by addition of NaOH to precipitate pectin (pH~3.5). Pectin precipitates were washed with propanol 70%, submitted to overnight drying at 65 °C and weighed.

In vitro antioxidant capacity was evaluated according to methods based on radical scavenging activity ABTS^•+^ (2,2′-azino-bis(3-ethylbenzothiazoline-6-sulfonate radical cation) and FRAP (Ferric Antioxidant Power) assays [26,28]. For all methods, the phenolic extracts were diluted with different volumes of ethanol: 1/0.5; 1/1.0; 1/1.5; 1/2.0 and 1/2.5 (volume of extract/volume of ethanol). The control solution was prepared by mixing ethanol and the respective standard solution.

Color was analyzed by a Hunter ColorFlex colorimeter (Reston, VA, USA) with D65 standard illumination and 10° observer angle. CIE L*a*b* color coordinates were converted to chroma (c*) and hue angle (h).

Microstructures of CH samples were observed by a JEOL JSM-5510 scanning electron microscope (SEM) (JEOL, Tokyo, Japan) at magnifications of 1000×. Coffee husk flours were dehydrated in an oven at 40 °C, for 24 h, and placed on SEM specimen tubs with double adhesive tape and coated with a 10 nm gold layer.

One milligram of flour was mixed with 10 mg of KBr and the spectra collected by IRAffinity-1 FTIR Spectrophotometer (Shimadzu, Japan) with a DLATGS detector with a 4 cm^−1^ resolution. KBr was utilized as background spectrum. Readings were carried out in dry atmosphere at 20 °C. Diffuse reflectance (DR) measurements were performed using a Shimadzu diffuse reflectance sampling accessory (DRS8000A).

Swelling capacity (SWC), water holding capacity (WHC), and oil holding capacity (OHC) were determined according to Resende et al. [28]. For SWC determination, 1 g of sample was combined with 100 mL of distilled water. Suspension was incubated at 230 rpm for 120 min and set for decantation for 18 h. The volume occupied by the samples was recorded and expressed as mL/g of sample. For WHC and OHC determinations, 25 mL of distilled water or soybean oil were mixed to 1 g of sample. The mixture was stirred at 150 rpm for 18 h and centrifuged at 3500× *g* rpm for 30 min. The supernatant was discarded, and the sample weighed. WHC and OHC were expressed as g water or oil/g of sample. SWC and WHC were also evaluated at 37 °C in isotonic solutions (NaCl 0.9% *m/v*), simulating conditions inside the stomach (pH = 1.5), duodenum (pH = 8.5) and also in pH = 7 in order to verify the effect of surrounding medium on hydration properties of CH samples.

### 2.8. Statistical Analysis

Analyses were performed in technical triplicates. Data were statistically analyzed by ANOVA and Tukey (*p* < 0.05) methods by IBM SPSS software (version 19). The normality of data was verified using the Shapiro–Wilk test.

## 3. Results

### 3.1. Phenolic Compounds

Data for contents of extractable (TEP) and non-extractable phenolic (NEP), chlorogenic acid (CGA), protocatechuic acid (APC), caffeine. trigonelline, and for bioaccessibility of phenolics in coffee husks (CH) are shown in Table 1. CH2 and CH2b samples presented lower TEP values than CH1 and CH1b, indicating coffee parchment contains lower contents of TEP than the outer skin and pulp. High concentration of phenolics in the outer skin is expected since the primary function of these metabolites is protection against external threats [26]. Thus, CH1 samples could be classified as a product with high content of phenolics (>500 mg GAE/100 g) [29], comparable to other fruits by-products such as buriti peels (785.1–934.6 mg GAE/100 g) [28]. Blanched samples presented lower TEP values than unblanched ones. Blanching is a pretreatment used to inactivate polyphenoloxidase enzymes responsible for the oxidation of phenolic compounds, thus favoring the maintenance of their antioxidant potential. However, it also favored the degradation of TEP compounds in CH, indicating these phenolic compounds were sensitive to the temperature applied during blanching [28].

Blanched CH samples presented NEP values higher than those of unblanched samples, confirming that polyphenoloxidases could be inactivated by heat treatment, preserving NEP. Also, these compounds were more strongly bound to the plant matrix, being somewhat protected from the high temperature during blanching. The increase of NEP contents with blanching is attributed to the removal of soluble solids, which decreased the dry basis mass. NEP contents of all samples were significantly higher than those of other fruit by-products, such as pequi (346.84 mg/100 g), apple (1278.7 mg/100 g), kiwi (1522.0 mg/100 g), melon (316.2 mg/100 g), nectarine (1797.5 mg/100 g), pear (721.1 mg/100 g), and watermelon (305.3 mg/100 g) [26,30]. In addition, NEP contents for blanched samples were comparable to that for grape bagasse (2716 mg/100 g), which was considered a by-product with a high content of non-extractable phenolics [22].

**Figure 1:**
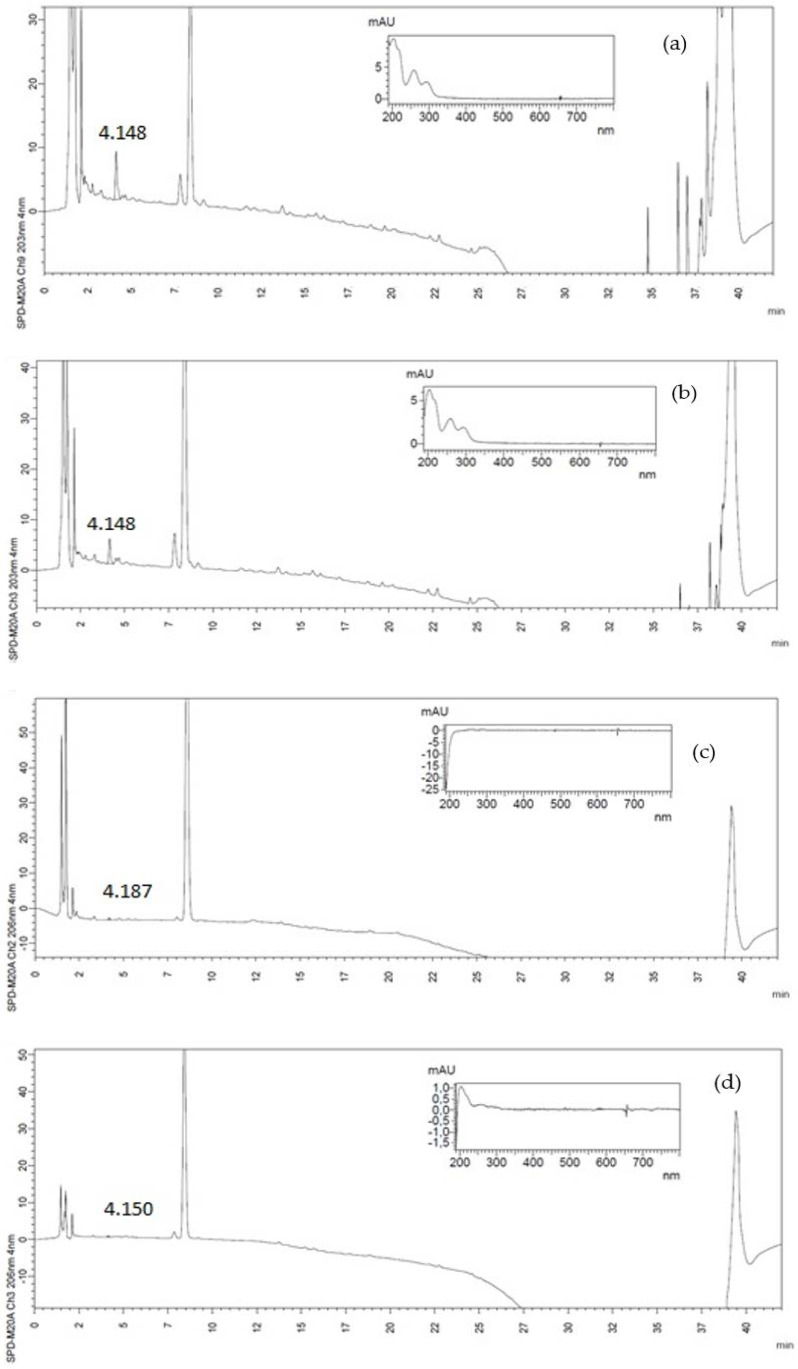
Protocathecuic acid (PCA) chromatograms and ultraviolet–visible (UV-Vis) spectra for (**a**) CH1, (**b**) CH1b, (**c**) CH2 and (**d**) CH2b, respectively at 206 nm.

**Figure 2:**
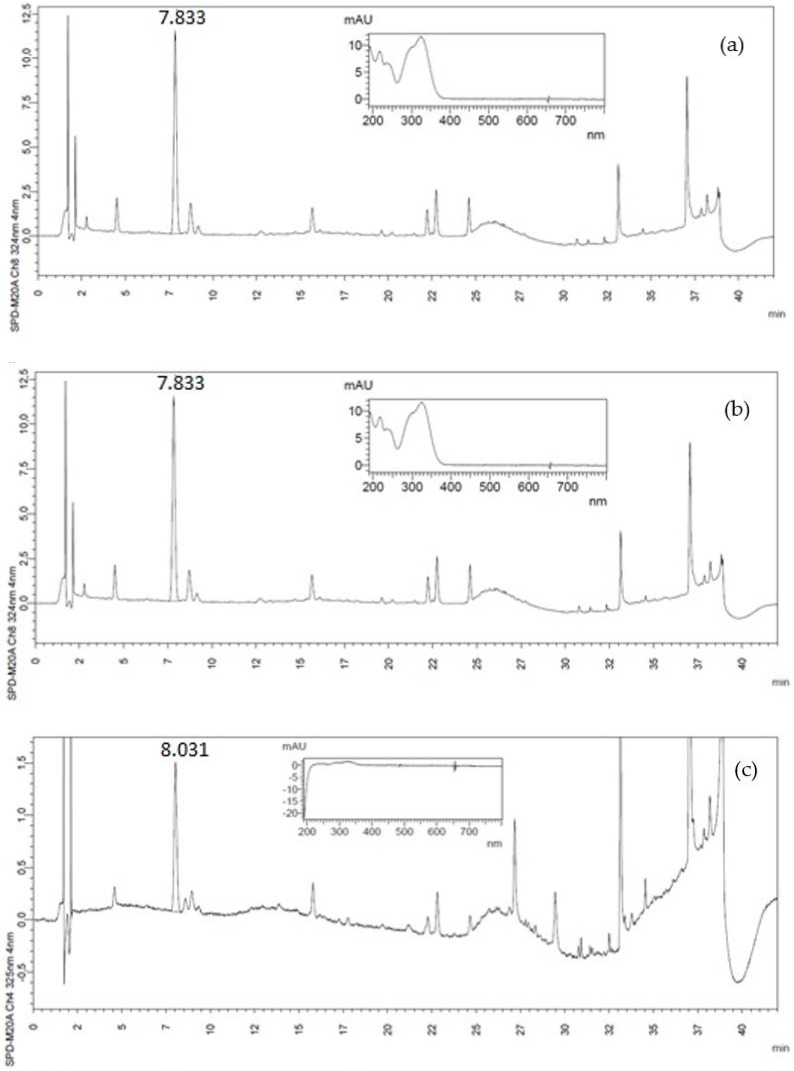

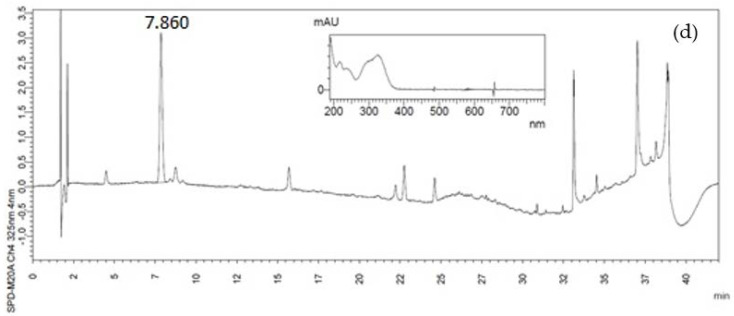
Chlorogenic acid (CGA) chromatograms and spectra for (**a**) CH1, (**b**) CH1b, (**c**) CH2 and (**d**) CH2b, respectively at 325 nm.

Chlorogenic (CGA) and protocatechuic (PCA) acids are phenolic compounds that exhibit a variety of biological functions in the human body, such as anti-inflammatory, antioxidant, and anticarcinogenic bioactivities [31,32]. CGA was identified in all CH samples, whereas only traces of PCA were detected in CH2 samples (Table 1, associated chromatograms in Figure 1 and Figure 2, respectively). CGA contents were considerably higher than those of other phenolics in coffee by-products [33]. Performing qualitative and quantitative PCA analyzes in complex matrices, such as foods, is difficult due to its low concentration and coelution with interferents in HPLC analysis [31]. CH2 and CH2b samples presented CGA values significantly lower than CH1 and CH1b, and blanched samples presented higher values of CGA than unblanched ones. These compounds were also preserved due to inactivation of polyphenoloxidases during the blanching step. CGA can also form complexes with carbohydrates or be bound to proteins present in the CH matrix, increasing their thermal stability and hindering degradation [34]. PCA content for CH1b was lower than for CH1, suggesting this compound was more susceptible to degradation during blanching. Despite this, CH1 samples showed higher PCA contents than other fruits, such as apple (1.3 mg/100 g), peach (1.24 mg/100 g), and nectarine (1.4 mg/100 g) [21].

Simulated buccal, gastric, and duodenum digestion of CH were performed to evaluate polyphenols stability under physiological conditions. Most studies have determined the bioactive properties of phenolic compounds without considering their physicochemical modifications after being submitted to digestive conditions. Besides, assessing the bioaccessibility of bioactive compounds is also important to further evaluate their actual availability in the human body. Bioaccessibility values of phenolics in coffee husks ranged from 64.10% to 70.76%, indicating most phenolic compounds in CH flours were bioaccessible. The interactions of polyphenols with lipids, proteins and cell wall polysaccharides (CPS) can significantly affect the respective bioaccessibility, bioavailability, and bioefficacy [35,36]. Although polyphenols can interact with lipids by hydrophobic bonding, and hydrogen and covalent bonds [36], the content of lipids in the coffee husks studied herein is rather low and interactions of such types might be considered negligible. Investigations of interactions of polyphenols with proteins in cell walls by other researchers have led to the conclusion that proteins do not cause significant effects on polyphenol adsorption under the studied conditions [35]. Therefore, it can be safely assumed that the bioaccessibility of the phenolic compounds in our study was mostly determined by interactions of polyphenols with cell wall polysaccharides. Strong evidence indicates that CPS–polyphenol interactions can be significantly affected by physicochemical characteristics of both constituents [35]. The chemical composition, molecular weight, degree of esterification, the type of side chains and branching ratios, and porosity of cell wall polysaccharides and the solubility, molecular weight, functional groups, and conformation of polyphenols are the major factors affecting the interactions of CPS and polyphenols. Benitez et al. [37], in their study of coffee parchment as a source of dietary fiber, determined that xylose was the predominant monosaccharide in the dietary fiber fraction of coffee parchment. It was also determined that galactomannans and arabinogalactans were not present in the parchment dietary fiber fraction since arabinose and galactose were not, therein, detected. Furthermore, cellulose was determined to be the second most abundant polysaccharide in coffee parchment. Pectic polysaccharides, comprised solely of uronic acids, were the less abundant polysaccharides (4–7% of coffee parchment composition) and the majority was linked to the cellulose matrix. Lignin content of coffee parchment was also significant (27–32%). Reichembach and Petkowicz [38] investigated coffee pulp as a source of pectin and concluded that pectin content in the alcohol insoluble fraction was about 14.6%. Cellulose and hemicelluloses have been deemed to present relatively lower affinities for polyphenols than pectins in solution or suspension [35]. Interactions of CPS with polyphenols most commonly occurs through electrostatic forces (i.e., non-covalent bonds) and hydrogen bonds as Van der Waals forces between hydroxyl groups of the phenolic compounds and different functionalities of the polysaccharides [36]. Although pectin may be present twice as much in coffee pulp and peel than in parchment [37,38] and the amount of negatively charged arabinoxylans probably twice as much in parchment than in coffee pulp and peel (see Section 3.3), any differences in interactions of these polysaccharides with the polyphenols might have been compensated by the distinct proportions of pulp and peel and parchment in samples CH1 and CH2 (or CH1b and CH2b), culminating in rather close values for bioaccessibility of polyphenols for both types of samples. Any attempt to further explain the small differences in bioaccessibility of polyphenols (4 to 5 percentage points) for the CH1 and CH2 samples herein studied would be pure speculation since no rigorous study was carried out on the specific factors that affect CPS-polyphenols interactions.

### 3.2. Trigonelline and Caffeine

Caffeine and trigonelline are important bioactive compounds in coffee beans [4]. They are soluble in water; therefore, a considerable amount of these components can be leached in industrial processes that use water, such as in the herein blanching step. The loss of caffeine and trigonelline in the blanching step depends on processing time and water temperature, which must be less than the values applied in their extraction processes. Herein, the samples were exposed to hot water for only 1 min; however, significant losses of both compounds occurred (Table 1). Loss of trigonelline (47.4% for CH1 and 51.2% for CH2) was significantly higher than the loss of caffeine (15.5% for CH1 and 40% for CH2), since trigonelline is a zwitterion readily soluble in water, whereas caffeine is moderately soluble in water with a slight increase in solubility with temperature and with formation of complexes with chlorogenic acid. Other studies have determined caffeine and trigonelline contents in green coffee to be 919 mg/100 g and 1029.9 mg/100 g, respectively [1,25]. Thus, caffeine and trigonelline contents in Arabica coffee husks amounts to more than half of those in coffee beans. Even with the loss of trigonelline after blanching, the processed by-products still present high trigonelline contents when compared to other relevant sources, such as fenugreek seeds (0.17–0.28%). Trigonelline is an important bioactive compound to humans due to its neuroprotective, hypolipidemic, antidiabetic, antihypertensive, and kidney, liver and heart protective activities [39].

### 3.3. Characterization of Polysaccharides

The identification of monosaccharides is relevant for determining the structure of the polysaccharides present in coffee husks. The relative molar percentages (% mol) of monosaccharides identified in the CH samples are presented in Table 2. In general, the samples comprised mostly of the outer skin (CH1 and CH1b) presented arabinose, mannose, and glucose contents higher than those comprising mostly parchment (CH2 and CH2b). No differences in rhamnose and galactose contents were observed among them. These monosaccharides in the by-products can indicate the presence of polysaccharides similar to those present in coffee beans, such as galactomannans (galactose, mannose, and traces of glucose and arabinose) and arabinogalactans (arabinose, galactose, and traces of rhamnose) [40,41].

All samples showed high percentages of xylose, indicating the presence of xylans and xyloglucans in CH. Xyloglucans also have glucose and might have galactose in their structure. Thus, samples that showed higher percentages of xylose and lower percentages of glucose and galactose (CH2 and CH2b) probably contain more xylans (or arabinoxylans) and fewer xyloglucans (xylose and glucose). Only arabinose (19.93 mol%), mannose (4.43 mol%), galactose (60.27 mol%) and glucose (15.37 mol%) were found in spent coffee grounds [41], and this fact shows that xylose is present only in the husks and is thus used to detect and identify adulteration of roasted and ground coffee with coffee husks.

Blanching caused an increase in glucose percentages in CH samples. This treatment causes softening of the vegetable matrix, probably due to hydrolysis of polysaccharides. Thus, glucose in CH samples can be released after hydrolysis of polysaccharides such as hemicellulose, pectin, and cellulose at high temperatures. In general, samples showed traces of myo-inositol, and these results were similar to the contents of myo-inositol in fresh pulp of lemon, tangerine, orange, and grape (0.6–2.1%) [42]. Myo-inositol is a glucose isomer abundant in cereals and fruits, and deficient in foods of animal origin [43]. Thus, samples with high glucose contents (CH1) presented myo-inositol contents higher than those with low glucose contents (CH2) (Table 2). Studies have shown that myo-inositol presented bioactivities in humans, such as reducing rates of gestational diabetes, and preventing depression and panic syndrome [44].

**Figure 3:**
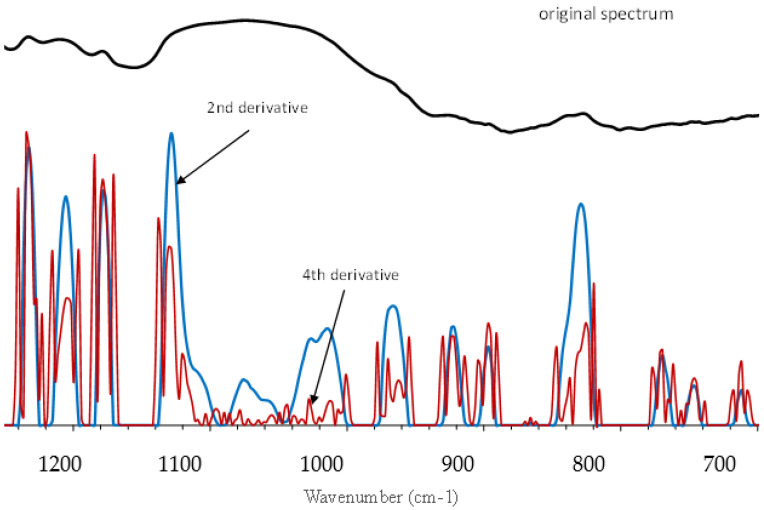
Fourier transform infrared (FTIR) spectrum for blanched coffee husks (black line) and its second (blue line) and fourth (red line) derivatives.

The FTIR spectrum of CH2b in the range of 1250 to 700 cm^−1^ (polysaccharide fingerprint region) and its second and fourth derivatives (absolute values of negative peaks) are presented in Figure 3 and, in conjunction with the monosaccharide composition results, is herein employed to tentatively characterize the polysaccharides present in coffee husks. This region is characterized by broad bands in the original spectrum, making it difficult to analyze. The results for monosaccharide composition demonstrated that coffee husks flour is comprised of different hemicellulotic and pectic polysaccharides, thus, overlapping and shifting of a few band peaks is expected since most of these polysaccharides have common types of linkage and functional groups and they interact with each other in the cell wall matrix. However, characteristic absorption bands of a specific polysaccharide or respective individual components allow for its discrimination from the others. Thus, the second derivative of the spectrum was taken and analyzed. The second derivative has the advantage of resolving the peaks that are lumped into the broad bands in the original spectrum and the wavenumbers of the local maxima of its negative peaks coincide with the wavenumbers of the local maxima of the peaks in the original spectrum. The fourth derivative was also taken since there were still a few overlapping peaks in the second derivative. The presence of pectic polysaccharides in CH2b is corroborated by the peak maxima at 1233, 1138, 1107, 1070, 1016, 897 and 829 cm^−1^ [45,46,47]. There are no bands at the vicinity of 878 cm^−1^ (characteristic of mannuronic acid) and rhamnose was detected in reasonable amounts in the samples, hence, it can be safely inferred that the pectic polysaccharides in coffee husks are of the rhamnogalacturonan type, since the bands at 1107 and 1016 cm^−1^ are characteristic of homogalacturonan content in pectin [48] and the peak at 829 cm^−1^ is attributed to out of plane hydroxyl vibrations characteristic of α-linkage between (1→4) galacturonic acid units in the backbone of pectin [47]. Also, the bands at 1233 and 1016 cm^−1^ are, respectively, attributed to bending of hydroxyl in pyranose ring of pectin and to C–O and C–C stretching vibrations in pectin [47]. The presence of cellulose is corroborated by the absorption bands 1204, 1103, 1095, 1055, 1028, 1000 and 897 cm^−1^, with the wavenumber 897 cm^−1^ corresponding to C1 group frequency or ring frequency, characteristic peak of β-glycosidic linkages in sugar units, 1000 cm^−1^ characteristic of C–O and C–C stretching in cellulose [47] and 1028 cm^−1^ attributed to stretching vibrations of β-(1→4) glycosidic linkage of glucans [48]. Arabinogalactans type II are characterized by the absorption bands 1138, 1078, 1065, 1044, 1020, 978, 964 and 891 cm^−1^, with 1044 cm^−1^ attributed to steric interaction between side-chain arabinofuranosyl units and galactopyranosyl backbone at 1078 cm-1 in arabinogalactan type II [48], 1138 and 978 cm^−1^ attributed to α-(1→3) glycosidic links of side-chain arabinose [46] and 891 cm^−1^ attributed to stretching vibration of anomeric C–H of β-galactopyranosyl units in the arabinogalactan backbone [49]. Galactomannans are characterized by the wavenumbers 1090, 1061, 1033, 897, 870, 816, 770, 747, 740 and 717 cm^−1^ with 1090 and 1061 cm^−1^ corresponding to stretching vibrations of C–O–C in β-(1→4) links of mannan polymers and 1033 cm^−1^ attributed to C–C bonds of mannose unit rings [50]. The band 870 cm^−1^ is characteristic of sidechain (1→6)-α-D-galactopyranosyl units in mannan backbone and the band 816 cm^−1^ attributed to (1→4)-β-D-mannopyranosyl units in the backbone of galactomannans [51]. The wavenumbers 770, 747, 740 and 717 cm^−1^ correspond to the skeletal bending of galactose rings and to C–O–C bending vibration separately in galactomannan glycosidic linkages [49]. The peaks at 1171, 1121, 1078, 1072, 1049, 1040, 955, 930 and 891 cm^−1^ are suggestive of the presence of xyloglucans and arabinoxylans, in which the higher frequencies at 1171, 1121 and 1040 cm^−1^ are characteristic of xylans [45], the wavenumber 930 cm^−1^ is attributed to ring vibration and 891 cm^−1^ attributed to stretching of the β-anomeric link in xyloglucans [47]. The presence of arabinoxylans is corroborated by the aforementioned peaks characteristic of xylans and the peaks at 1049 cm^−1^, associated with linear and branched (1→4)-β-xylan, and 955 cm^−1^, characteristic of disubstituted xylose residues in arabinoxylans [48].

In general, samples comprising mostly the outer skin and pulp (CH1) showed values for TEP, CGA, PCA, NEP, bioaccessibility of phenolics, trigonelline, and some monosaccharides contents (i.e., arabinose, mannose, glucose, and myo-inositol) higher than those of samples comprising mostly parchment (CH2), confirming its highest nutritional and bioactive potential as food ingredient. Despite the loss of some compounds (TEP, PCA, caffeine, and trigonelline) by heat treatment, others could be better preserved (NEP and CGA). However, significant amounts of TEP, PCA, caffeine, and trigonelline could be maintained even after blanching. Considering industrial production, the use of blanched husks as a food ingredient would be more favorable for product conservation due to its microbiological load reduction and enzyme inactivation. For these reasons, CH1b was selected to be chemically characterized and evaluated for its technological potential, antioxidant capacity, and microstructure.

### 3.4. Chemical, Physical, and Technological Properties

The proximal composition of CH1b (Table 3) was determined and respective values for fat (5.01 ± 0.12 g/100 g), ash (5.20 ± 0.08 g/100 g), crude protein (10.30 ± 0.01 g/100 g) and TDF (65.83 ± 1.68 g/100 g) were higher than those determined for other fruit by-products, such as buriti [28] and pequi [26] peels. Moreover, TDF values reported for coffee pulp and husks by Murthy and Naidu [52] were also lower than the herein determined. These variations are expected due to the inherent differences in coffee cultivars and in the techniques used in the by-product’s treatment. Lower TDF contents determined by Murthy and Naidu [52] for coffee pulp and husks (28 ± 43 g/100 g) were probably the result of fiber degradation caused by high pressures and high temperatures applied over a long period (around 30 min). CH1b showed similar characteristics of commercialized fiber products (TDF above 50% and moisture below 9%) even after blanching treatment. Thus, it can be an interesting alternative to be used as a dietary fiber source in the food industry.

The soluble and insoluble characteristics of fibers affect their technological functionality and physiological effects [28]. IDF content of CH1b (58.69 g/100 g) is also high compared to those of other by-products (pequi peel: 30.3–33.94 g/100 g and buriti peel: 47.91–49.21 g/100 g) [26,28]. A high IDF content may be associated with a large amount of total non-digestible carbohydrates and low available carbohydrate content. CH1b mostly comprises IDF (>50%); therefore, it might present pronounced effects on intestinal regulation and stool volume since these effects are associated with the consumption of products with high contents of insoluble fibers. SDF is also attractive due to its capacity to decrease plasma cholesterol and glycemic response, and its prebiotic action [53]. SDF of CH1b (7.97 ± 0.36) mostly comprises pectin (~83%) and its content is comparable to those of apple, peach, and tomato (~6−7.5 g/100 g) [53]. Pectin is a soluble fiber widely applied as a gelling agent and stabilizer in food matrices.

CH1b presented high antioxidant activity (755.9 ± 47.97 µmol Trolox/g; 175.78 ± 0.49 µmol Fe_2_SO_4_/g). The antioxidant activities of CH1b determined by different techniques were higher than or similar to those of many matrices known as a potent antioxidant, such acai (220 ± 32.9 µmol Fe_2_SO_4_/g) and spent coffee grounds (180 ± 57 µmol Trolox/g) [54,55]. Such antioxidant activity of CH1b can be attributed its high contents of polysaccharides [56] and of extractable phenolics (904.74 mg GAE/100 g).

The technological properties that render a by-product suitable for utilization as food ingredient were determined for CH1b and are presented in Table 4. Luminosity of CH1b (L = 44.93 ± 0.18) was low compared to other by-products obtained from fruit peels (buriti: 53.13 < L* < 62.38 and pequi: 55.2) [54,55]. The low luminosity of CH1 can be partially attributed to the degradation of anthocyanins by the action of enzymes (peroxidases and polyphenoloxidases), liberated by the damaged cells of the outer skin and pulp during handling and drying, or by other oxidizing agents, such as oxygen [18]. Despite this, coffee husks flours were clearer than other by-products flours [54,55]. This characteristic is desirable in applications where food product color should not be changed by adding an ingredient. Chroma (c*) and hue angle (h) values for CH1b are in the same range of those for other fiber-rich by-products flours [54,55], with h° indicating a predominance of yellow over red (>45°).

WHC represents the water quantity that remains in hydrated flours after application of an external force and SWC indicates how much the fiber matrix increases in size when water is absorbed. SWC value for CH1b (8.75 ± 0.35 mL/g) was higher than those of by-products of buriti (3.7 mL/g), mango (4.6 mL/g), passion fruit (7.2 mL/g), pineapple (6.6 mL/g), and guava (1.4 mL/g), whereas WHC value (4.08 g/g) was higher than those of buriti blanched peel (1.14 g/g) and pequi peel (3.74–3.98 g/g) [54,55,57]. High WHC and SWC values are usually associated with high levels of SDF because soluble fibers present better hydration properties than insoluble ones [58]. In human bodies, fiber swelling promoted by hydration increases fecal bulking and decreases its transit time, which can prevent colon cancer [59].

Unlike water hydration properties, oil retention capacity is related to the presence of hydrophobic groups in the food matrix. Proteins are characterized by presenting hydrophilic and hydrophobic functional groups. These hydrophobic groups can be associated physicochemically with non-polar chains of the oil and promote their retention. Therefore, the higher the protein content, the greater the oil holding capacity. As the protein content of CH1b was higher than those of other fruit peel by-products, OHC value for CH1b was consequently higher [54,55]. High OHC values favor flavor retention ability and emulsification capacity of by-products, preclude loss of fat during processing, and bring health benefits associated with the reduction of plasma cholesterol [59].

Regarding SWC and WHC results for samples evaluated under physiological conditions (isotonic solutions at 37 °C at different pH), hydration properties of CH1b increased with increasing pH from 1.5 to 8.5. SWC values were 6.87 ± 0.03, 9.02 ± 0.37 and 10.60 ± 0.52 mL/g respectively for pH 1.5, 7.0 and 8.5; and WHC values were 4.12 ± 0.02, 4.16 ± 0.01 and 4.19 ± 0.04 g H_2_O/g respectively for pH 1.5, 7.0 and 8.5. The progressive ionization of the chains of the different polysaccharides present in the CH1b, mostly pectin (6.58 ± 0.01 g/100 g; 83% of SDF) contributed to the increase of its hydration properties. Carboxylic groups and carboxylic esters are the main functional groups of pectin. Thus, the ionization of carboxylic groups in alkaline medium led to an expansion of the pectin structure due to effects of electrostatic repulsion between negative charges present in the chains [60].

**Figure 4:**
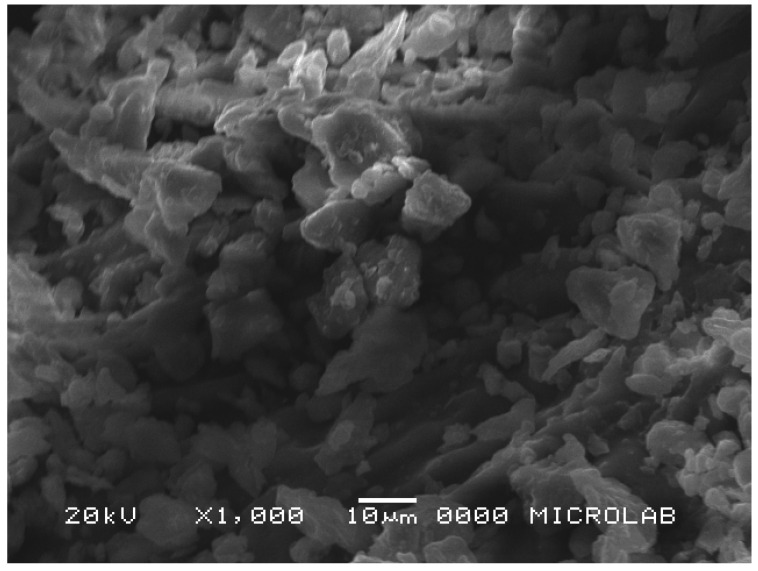
Scanning electron microscopy image of blanched coffee husks comprised mostly of outer skin (CH1b).

The morphology of CH1b was observed by scanning electron microscopy (Figure 4). CH1b exhibited irregular surfaces with fragments or symmetric particles ranging in size from 5 to 40 µm. The particle size is the most important physical parameter defining flour behavior. Smaller particle sizes are expected to improve the coarse sensory properties of the final products in which the flours are used as ingredients. The particle sizes herein determined for CH1b flours are in the same range of those of wheat bran particles (0.5–100 µm) that were successfully applied by Song et al. [61] in food products to replace wheat flour. Considering the antioxidant capacity and all technological properties evaluated, CH1b flour showed great potential for use as a food ingredient.

## 4. Conclusions

The chemical composition of coffee husks was investigated. Blanching of coffee husks had a positive effect on the bioactive potential of these by-products demonstrating their potential for use as a functional food ingredient. Coffee husks (CH) comprising mostly outer skin and pulp (CH1) showed higher bioactive potential and bioaccessibility of phenolics than CH comprising mostly parchment (CH2). NEP contents were higher than TEP ones, indicating most of the phenolics of CH were trapped in the plant matrix. Blanching treatment led to the loss of some compounds (TEP, PCA, caffeine, and trigonelline), but others (NEP and CGA) were better preserved, depending on the level of interaction of the compounds with the matrix and their thermal stability. The polysaccharide fraction was characterized with cellulose, pectic polysaccharides, arabinogalactans type II, galactomannans, xyloglucans and arabinoxylans being tentatively identified. The blanched CH1 sample (CH1b) showed significant potential to be used as ingredient in food products since it had similar characteristics of commercialized fiber products, predominantly yellow color (h = 67.28) and small sized particles (5–40 µm). Besides, its values for antioxidant, hydration properties, and oil holding capacity were higher than those of other fruit by-products, which were associated with the presence of phenolic compounds, insoluble fibers, and proteins, respectively. Results confirmed the high nutritional and bioactive potential of coffee husks.

## Figures and Tables

**Table 1:** Extractable (TEP) and non-extractable (NEP) phenolics, bioaccessibility of phenolics (TEP %), chlorogenic acid (CGA), protocatechuic acid (PCA), caffeine and trigonelline in coffee husks.

Sample	TEP (mg GAE/100 g)	NEP (mg/100 g)	CGA (mg/100 g)	PCA (mg/100 g)	Bioaccessibility TEP %	Caffeine (mg/100 g)	Trigonelline (mg/100 g)
CH1	983.05 ± 32.68 ^a^	2025.85 ± 55.68 ^c^	121.55 ± 0.18 ^b^	28.24 ± 0.18 ^a^	70.76	618.10 ± 6.66 ^b^	542.80 ± 6.54 ^a^
CH1b	904.74 ± 9.47 ^b^	2703.33 ± 10.73 ^a^	174.27 ± 0.26 ^a^	14.42 ± 0.01 ^b^	68.64	522.09 ± 0.62 ^c^	285.58 ± 4.83 ^b^
CH2	274.77 ± 6.35 ^c^	1837.77 ± 23.73 ^d^	17.19 ± 0.13 ^d^	-	65.74	696.22 ± 4.86 ^a^	246.21 ± 2.56 ^c^
CH2b	196.31 ± 12.54 ^d^	2387.34 ± 32.88 ^b^	43.09 ± 0.16 ^c^	-	64.10	418.13 ± 0.65 ^d^	120.16 ± 1.20 ^d^

Mean ± standard deviation (*n* = 3). Different letters in the same column indicate that values are significantly different (*p* > 0.05).

**Table 2:** Relative molar percentages (RMP) of monosaccharides in coffee husks (CH).

Monosaccharides	CH1 (% mol)	CH1b (% mol)	CH2 (% mol)	CH2b (% mol)
Rhamnose	3.04 ± 0.60 ^a^	2.98 ± 0.52 ^a^	2.66 ± 0.38 ^a^	2.46 ± 0.58 ^a^
Arabinose	25.24 ± 1.37 ^a^	25.17 ± 1.26 ^a^	17.91 ± 1.77 ^b^	16.14 ± 1.77 ^b^
Xylose	32.92 ± 1.46 ^b^	23.69 ± 1.17 ^c^	55.53 ± 1.89 ^a^	53.66 ± 1.87 ^a^
Mannose	9.67 ± 0.90 ^a^	10.00 ± 1.10 ^a^	5.33 ± 0.96 ^b^	4.47 ± 0.86 ^b^
Galactose	11.91 ± 2.40 ^ab^	13.69 ± 1.78 ^a^	9.59 ± 2.32 ^b^	10.52 ± 1.39 ^b^
Glucose	15.84 ± 1.32 ^b^	22.92 ± 1.28 ^a^	8.68 ± 0.92 ^d^	12.74 ± 1.23 ^c^
Myo-inositol	1.38 ± 0.12 ^a^	1.54 ± 0.17 ^a^	0.30 ± 0.04 ^b^	0

Mean ± standard deviation (*n* = 3). Different letters in the same line indicate that values are significantly different (*p* > 0.05).

**Table 3:** Centesimal composition of CH1b.

Centensimal Composition								
Moisture (g.100 g^−1^)	Protein (g.100 g^−1^)	Fat (g.100 g^−1^)	Ash (g.100 g^−1^)	TDF (g.100 g^−1^)	IDF (g.100 g^−1^)	SDF (g.100 g^−1^)	Pectin Content (g.100 g^−1^)	Pectin in SDF (%)
5.50 ± 0.05	10.03 ± 0.01	5.01 ± 0.12	5.20 ± 0.08	65.83 ± 1.68	58.69 ± 0.45	7.97 ± 0.36	6.58 ± 0.01	82.91 ± 2.44

Mean ± standard deviation (*n* = 3). TDF—Total dietary fiber; IDF—Insoluble dietary fiber; SDF—Soluble dietary fiber.

**Table 4:** Technological properties and antioxidant capacity of CH1b.

Technological Properties						Antioxidant Capacity	
Luminosity (L)	Hue Angle (h)	Color Saturation (c)	OHC (g oil. g^1^)	SWC (mL.g^−1^)	WHC (g water.g^−1^)	ABTS (µmol Trolox.g^−1^)	FRAP (µmol Fe_2_SO_4_. g^−1^)
44.93 ± 0.18	67.28 ± 0.09	25.79 ± 0.10	5.21 ± 0.15	8.75 ± 0.35	4.08 ± 0.05	755.9 ± 47.97	175.78 ± 0.49
				(pH 1.5) 6.87 ± 0.03 ^a^	(pH 1.5) 4.12 ± 0.02 ^a^		
				(pH 7.0) 9.02 ± 0.37 ^b^	(pH 7.0) 4.16 ± 0.01 ^b^		
				(pH 8.5) 10.60 ± 0.52 ^c^	(pH 8.5) 4.19 ± 0.04 ^b^		

Mean ± standard deviation (*n* = 3). Different letters in the same line indicate that values are significantly different (*p* > 0.05). OHC—Oil holding capacity; SWC—Swelling capacity; WHC—Water holding capacity.

## Data Availability

The data presented in this study are available on request from the corresponding author.
